# Genetic Basis and Clonal Population Structure of Antibiotic Resistance in *Campylobacter jejuni* Isolated From Broiler Carcasses in Belgium

**DOI:** 10.3389/fmicb.2018.01014

**Published:** 2018-05-17

**Authors:** Mohamed Elhadidy, William G. Miller, Hector Arguello, Avelino Álvarez-Ordóñez, Alexandra Duarte, Katelijne Dierick, Nadine Botteldoorn

**Affiliations:** ^1^Department of Bacteriology, Mycology and Immunology, Faculty of Veterinary Medicine, Mansoura University, Mansoura, Egypt; ^2^University of Science and Technology, Zewail City of Science and Technology, Giza, Egypt; ^3^Produce Safety and Microbiology Research Unit, Agricultural Research Service, U.S. Department of Agriculture, Albany, CA, United States; ^4^Genomics and Animal Breeding, Department of Genetics, Faculty of Veterinary Science, University of Córdoba, Córdoba, Spain; ^5^Department of Food Hygiene and Technology, Institute of Food Science and Technology, University of León, León, Spain; ^6^Laboratory of Food Microbiology and Food Preservation, Department of Food Safety and Food Quality, Faculty of Bioscience Engineering, Ghent University, Gent, Belgium; ^7^National Reference Laboratory for Campylobacter, Scientific Institute of Public Health (WIV-ISP), Scientific Service: Foodborne Pathogens, Brussels, Belgium

**Keywords:** broiler, antibiotic resistance, resistance genes, mutations, MLST

## Abstract

Human campylobacteriosis is the leading food-borne zoonosis in industrialized countries. This study characterized the clonal population structure, antimicrobial resistance profiles and occurrence of antimicrobial resistance determinants of a set of *Campylobacter jejuni* strains isolated from broiler carcasses in Belgium. Minimum inhibitory concentrations (MICs) against five commonly-used antibiotics (ciprofloxacin, nalidixic acid, tetracycline, gentamicin, and erythromycin) were determined for 204 *C. jejuni* isolates. More than half of the isolates were resistant to ciprofloxacin or nalidixic acid. In contrast, a lower percentage of screened isolates were resistant to gentamicin or erythromycin. *C. jejuni* isolates resistant to ciprofloxacin and/or nalidixic acid were screened for the substitution T86I in the quinolone resistance determining region (QRDR) of the *gyrA* gene, while *C. jejuni* isolates resistant to tetracycline were screened for the presence of the *tet(O)* gene. These resistance determinants were observed in most but not all resistant isolates. Regarding resistance to erythromycin, different mutations occurred in diverse genetic loci, including mutations in the 23S rRNA gene, the *rplD* and *rplV* ribosomal genes, and the intergenic region between *cmeR* and *cmeABC*. Interestingly, and contrary to previous reports, the A2075G transition mutation in the 23S rRNA gene was only found in one strain displaying a high level of resistance to erythromycin. Ultimately, molecular typing by multilocus sequence typing revealed that two sequence types (ST-824 and ST-2274) were associated to quinolones resistance by the presence of mutations in the gene *gyrA* (*p* = 0.01). In addition, ST-2274 was linked to the CIP-NAL-TET-AMR multidrug resistant phenotype. In contrast, clonal complex CC-45 was linked to increased susceptibility to the tested antibiotics. The results obtained in this study provide better understanding of the phenotypic and the molecular basis of antibiotic resistance in *C. jejuni*, unraveling some the mechanisms which confer antimicrobial resistance and particular clones associated to the carriage and spread of resistance genes.

## Introduction

*Campylobacter* spp., and in particular *Campylobacter jejuni*, are considered among one of the most prevalent zoonotic foodborne pathogens associated with sporadic diarrhea in humans. In the European Union, human campylobacteriosis is by far the most frequent foodborne infection, with more than 200,000 confirmed human cases/year (EFSA, 2015). Most of the cases are self-limiting and include symptoms such as fever, abdominal cramps and watery to bloody diarrhea. In severe cases, infection might lead to post-infectious neurological complications such as Guillain-Barré syndrome and Miller-Fisher syndrome (Humphrey et al., 2007). Consumption of contaminated poultry is the main source of human *Campylobacter* infections (Kaakoush et al., 2015). In poultry, the pathogen colonizes the gut in relatively high concentrations but produces few or no clinical symptoms (Luangtongkum et al., 2006). These high numbers of bacteria can contaminate broiler carcasses during slaughtering, with the subsequent transmission of *Campylobacter* through the food chain (Jeffrey et al., 2001).

Frequent isolation of antimicrobial-resistant *Campylobacter* strains of food animal origin represents a public health threat, as these sources may act as an important vehicle for the transmission of resistant strains to humans, where the spectrum of antibiotics efficient for disease treatment will be limited (Aarestrup and Engberg, 2001). As poultry and poultry products are considered the main reservoir for *Campylobacter* food-borne transmission, the intensive use of antimicrobial agents for therapy, prophylaxis, or as growth promoters in poultry production may lead to the selection of resistant strains that can be transmitted to humans via contaminated food (Aarestrup and Engberg, 2001). The use of antibiotics as growth promoters was banned in Europe in 2006 based on the European Community directive 90/167/EEC. Furthermore, in 2007, the World Health Organization (WHO) made recommendations for limiting the routine use of antimicrobials in production animals (Collignon et al., 2009). All EU member states are required to monitor antimicrobial resistance (AMR) in broiler flocks. The European Surveillance of Veterinary Antimicrobial Consumption (ESVAC) illustrates recent efforts of the European Medicines Agency to develop a central database on the use of antimicrobial agents in animals (EMA, 2009). Indeed, different antimicrobial consumption monitoring programs aimed at mitigating the risk related to antimicrobial resistance have been established in some European Countries, and Belgium is currently developing a veterinary antimicrobial consumption monitoring system (www.belvetsac.ugent.be).

Serious concerns associated with the increasing isolation frequency of resistant *Campylobacter* spp. have prompted the investigation of the resistance mechanisms to these antimicrobials. In *Campylobacter*, high levels of resistance to quinolones and fluoroquinolones are mainly due to single point mutations in the DNA gyrase gene *gyrA*, especially a C257T mutation that results in a T86I substitution (Iovine, 2013). The main mechanism that confers high-level resistance to macrolides in *Campylobacter* is the occurrence of point mutations in the peptidyl encoding region of the 23S rRNA gene (Vacher et al., 2005). In addition, modifications of the L4 or L22 ribosomal proteins, mediated by point mutations in the *rplD* and *rplV* genes, respectively, and the presence of the 23S rRNA methyltransferase gene *ermB* have been also identified as resistance mechanisms for macrolides (Pérez-Boto et al., 2010; Wang et al., 2014; Florez-Cuadrado et al., 2017). Overexpression of the *CmeABC* multidrug efflux pump also works synergistically with these resistance mechanisms to confer resistance to the action of macrolides (Gibreel et al., 2005). Resistance to tetracycline, on the other hand, is mediated by the ribosomal protection protein TetO that is encoded by the *tet(O)* gene, located on either the chromosome or on conjugatable plasmids (Gibreel et al., 2004; Wu et al., 2014).

MLST locus alleles have been previously used as a successful source-attribution model and in assigning genomes to host reservoir (Pascoe et al., 2017). In addition to the resistance mechanisms described above, several reports have previously highlighted the association of particular sequence types with certain antimicrobial resistance phenotypes in Europe, suggesting potential clonal expansion of antimicrobial resistance phenotypes in recent years (Habib et al., 2009; Cody et al., 2012; Kittl et al., 2013; Kovač et al., 2014, 2015; Klein-Jöbstl et al., 2016).

The aim of this study is to determine the frequency of antimicrobial resistance in a subset of 204 *C. jejuni* isolates recovered from broiler carcass swabs in Belgium, and to investigate the molecular mechanisms of resistance and the possible associations between antimicrobial resistance profiles and certain MLST genotypes.

## Materials and methods

### Bacterial strains and growth conditions

A collection of 204 *C. jejuni* isolates were obtained between 2006 and 2015 from broiler carcass samples collected from different slaughter lines according to the ISO 10272-1 method (ISO 10272*-*1*:*ISO, 2006). All isolates were subcultured from −80°C frozen stocks onto Columbia agar (Oxoid, United Kingdom) with 5% horse blood (Sigma-Aldrich, United Kingdom). Plates were incubated at 41.5 ± 1°C within a gas jar under microaerobic conditions (6% O_2_, 7% CO_2_, 7% H_2_, and 80% N_2_), provided by the Anoxomat (Mark II System, The Netherlands).

### Phenotypic antibiotic resistance profiling

Testing for resistance to ciprofloxacin, erythromycin, gentamicin, nalidixic acid and tetracycline was carried out by the standard broth microdilution method, using the commercial diagnostic test for *Campylobacter* minimum inhibitory concentrations (MICs) (Sensititre® plates; Sensititre *Campylobacter* plate–EUCAMP, Trek Diagnostic Systems, UK) and following the manufacturer's instructions. Briefly, bacterial suspensions adjusted at approximately 1.5 × 10^8^ CFU/ml (equivalent to 0.5 McFarland standard) were inoculated into 96-well microtiter plates containing Mueller–Hinton broth (Oxoid, UK) supplemented with 5% defibrinated horse blood. Following bacterial inoculation, the Sensititre *Campylobacter* Plates were incubated under microaerobic conditions (Anoxomat, Mark II System, The Netherlands) at 37°C for 48 h. The MIC was read as the lowest concentration completely inhibiting visible growth. Resistance against antibiotics was interpreted using the epidemiologic cut-off values (ECOFFs), following the guidelines of the European Committee on Antimicrobial Susceptibility Testing (EUCAST, 2015) (www.eucast.org). *C. jejuni* strain ATCC 33560 was included as a quality control in the antimicrobial susceptibility determinations.

### Molecular characterization of antimicrobial resistance phenotypes

Resistance mechanisms were evaluated for quinolones and fluoroquinolones (nalidixic acid and ciprofloxacin, respectively), macrolides (erythromycin) and tetracycline. DNA was extracted from overnight bacterial cultures using a DNeasy Blood & Tissue Kit (Qiagen, Germany) according to the manufacturer's instructions. DNA was eluted in 100 μl of the kit elution buffer and stored at−20°C for further molecular analysis of resistance determinants. *C. jejuni* isolates displaying resistance to ciprofloxacin and/or nalidixic acid were screened for a point mutation that results in the T86I substitution in the quinolone resistance determining region (QRDR) of *gyrA*, using the mismatch amplification mutation assay (MAMA-PCR) as previously described (Zirnstein et al., 1999). *C. jejuni* isolates resistant to tetracycline were screened for the presence of the *tet(O)* gene, as previously described by Gibreel et al. (2004). Erythromycin-resistant isolates were characterized for five genetic loci potentially responsible for resistance: the 23S rRNA gene; the *rplD* and *rplV* 50S ribosomal subunit genes; the *ermB* gene; and the intergenic region between *cmeR* and *cmeABC*. The mismatch amplification mutation assay previously described by Alonso et al. (2005) was used to identify A2074C and A2075G point mutations in the 23S rRNA gene previously associated with high-level erythromycin resistance. Modifications in the L4 and L22 ribosomal proteins were determined using amplification and sequencing of the *rplD* and *rplV* genes, respectively, as reported elsewhere (Corcoran et al., 2006). The presence of the *ermB* gene among resistant strains was assessed previously described by Zhou et al. (2016). Finally, different polymorphisms in the regulatory region of *cmeABC* were screened by PCR amplification and sequencing of the intergenic region between the *cmeR* and *cmeA* genes. All protocols, primer sequences, amplification and sequencing conditions were employed as described by referenced authors.

### DNA sequence analysis

Amplification products generated were purified using the QIAquick PCR Purification kit (Qiagen, Germany). Purified amplicons were sequenced using the ABI PRISM BigDye terminator cycle sequencing kit (ver. 3.1; Life Technologies, Grand Island, NY) and standard protocols. DNA sequencing was performed on an ABI PRISM 3730 DNA Analyzer (Life Technologies), using POP-7 polymer and ABI PRISM Genetic Analyzer Data Collection and ABI PRISM Genetic Analyzer Sequencing Analysis software. Sequences were manually edited and then compared to those in the current databases using the BLAST suite of programs. Sequence alignments and SNP identification was performed using the Lasergene analysis package (v. 8.0; DNASTAR, Madison, WI).

### Analysis of genetic similarity among antimicrobial-resistant strains

The genetic similarity of antimicrobial-resistant isolates was analyzed using multilocus sequence typing (MLST) as previously described (Miller et al., 2005). Amplicons were sequenced as described above. Allele numbers, sequence types (STs) and clonal complexes (CCs) were assigned by submitting the DNA sequences to the *Campylobacter* PubMLST database website (https://pubmlst.org/campylobacter/) at the University of Oxford.

### Statistical analysis

Descriptive statistical analysis of the data was performed using R version 3.3.2 (R-project). Association among the different antimicrobial resistance determinants, and between AMR profiles and MLST STs and CCs were examined using Chi2 or Fisher's exact tests. The same statistical tests were used to establish changes or trends in susceptibility to the antimicrobials tested. Significance was established at α = 0.05.

## Results

### Antimicrobial resistance phenotypes

Fifty-eight (28.4%) isolates were pan-susceptible to all antimicrobials tested. The highest frequency of resistance was observed for ciprofloxacin (53.9%), followed by resistance to nalidixic acid (53.4%), and tetracycline (47%) (Table 1). In this study, almost all (95.5%) ciprofloxacin resistant isolates were cross-resistant to nalidixic acid. A low frequency of resistance was observed for gentamicin (6.9%) and erythromycin (4.9%). MIC tests yielded 18 different antimicrobial resistance patterns (Table 2).

**Table 1:** Antimicrobial resistance rates of *C. jejuni* from broiler carcasses.

**Rank**	**Class**	**Antimicrobial**	**Break points (mg/L)**	**No. of resistant isolates (%)**
I	Aminoglycosides	Gentamicin	>2	14 (6.9%)
	Macrolides	Erythromycin	>4	10 (4.9%)
	Quinolones and fluoroquinolones	Ciprofloxacin	>0.5	110 (53.9%)
		Nalidixic acid	>16	109 (53.4%)
||	Tetracyclines	Tetracycline	>1	96 (47%)

**Table 2:** Distribution of antimicrobial resistance patterns and MLST sequence types among *C. jejuni* from broiler carcasses.

**Antimicrobial resistance profile^a^**	**No. of isolates *n* (%)**	**Sequence types**
Sensitive	58 (28.4%)	ST-19 (1), ST-137 (2), ST-21 (4), ST-25 (1), ST-38 (1),
		ST-42 (1), ST-45 (7), ST-48 (2), ST-50 (7), ST-51 (1), ST-61 (1), ST-257 (2), ST-267 (1), ST-400 (1), ST-415 (1), ST-418 (1),
		ST-475 (1), ST-464 (1), ST-538 (1), ST-583 (3), ST-606 (1),
		ST-607 (1), ST-791 (1), ST-1044 (4), ST-1045 (1), ST-1326 (1),
		ST-2187 (1), ST-2314 (1), ST-3115 (1), ST-3293 (1),
		ST-3544 (1), ST-3548 (1), ST-4354 (1), ST-5018 (1), ST-5903 (1)
TET	25 (12.2%)	ST-48 (1), ST-50 (1),ST-141 (1), ST-257 (1), ST-464 (1), ST-879 (4), ST-2314 (1),ST-2324 (1),
		ST-2547 (1), ST-2641 (1), ST-2803 (1), ST-2844 (2), ST-3547 (1)
		ST-4602 (1), ST-5970 (1), ST-7953 (1), ST-7954 (1), ST-7960 (1), ST-8636 (1), ST-8637 (1),
		ST-8639 (1)
CIP	3 (1.5%)	ST-572 (1), ST-775 (1), ST-2132 (1)
ERY	2 (1%)	ST-42 (1), ST-4776 (1)
NAL	3 (1.5%)	ST-45 (1),ST-51 (1), ST-2027 (1)
GEN	2 (1%)	ST-45 (1), ST-122 (1)
Q	36 (17.6%)	ST-19 (3), ST-21 (4), ST-48 (3), ST-50 (1), ST-61 (1), ST-53 (1),
		ST-122 (1), ST-267 (1), ST-305 (1), ST-324 (1), ST-572 (2),
		ST-607 (1), ST-775 (1), ST-824 (4), ST-883 (1), ST-982 (1),
		ST-905 (1), ST-1519 (1), ST-1707 (1), ST-2258 (1), ST-4800 (1), ST-5018 (2), ST-8638 (1),
		ST-8640 (1)
NAL TET	2 (1%)	ST-51 (1), ST-2844 (1)
CIP TET	1 (0.5%)	ST-2882 (1)
ERY TET	1 (0.5%)	ST-1519 (1)
Q TET	58 (28.4%)	ST-19 (2), ST-21 (2), ST-44 (1), ST-46 (2), ST-48 (1), ST-50 (3),
		ST-51 (1), ST-141(1), ST-354 (2), ST-492 (1), ST-464 (5),
		ST-824 (1), ST-877 (1), ST-883 (2), ST-904 (2), ST-969 (1),
		ST-990 (3), ST-2135 (2), ST-2153 (1), ST-2252 (1), ST-2254 (3),
		ST-2274 (10), ST-2324 (1), ST-3015 (3), ST-3017 (1),
		ST-3155 (1), ST-3546 (1), ST-3720 (1), ST-3769 (1), ST-7487 (1)
Q GEN	2 (1%)	ST-305 (1), ST-824 (1)
**CIP GEN ERY**	1 (0.5%)	ST-2844 (1)
**CIP GEN TET**	2 (1%)	ST-474 (1), ST-3015 (1)
**NAL GEN TET**	1 (0.5%)	ST-45 (1)
**Q GEN TET**	1 (0.5%)	ST-606 (1)
**Q ERY GEN**	1 (0.5%)	ST-50 (1)
**Q ERY TET**	1 (0.5%)	ST-262 (1)
**Q ERY GEN TET**	4 (2%)	ST-48 (1), ST-354 (1), ST-824 (1), ST-8641 (1)

a *MDR strains are in bold and underlined. Q: CIP+NAL*.

### Genotypic characterization of antimicrobial resistance

Isolates that were resistant to ciprofloxacin and/or nalidixic acid were screened for T86I mutations in the *gyrA* gene. A total of 98 isolates that were simultaneously resistant to Cip and Nal harbored a C257T point mutation that resulted in a T86I substitution. Five isolates that showed simultaneous resistance to both antibiotics were negative for this mutation. Four Cip^r^ Nal^s^ isolates presented C257T point mutations in the QRDR. However, two Cip^r^ Nal^s^ isolates (cj1396, cj2940) had no C257T point mutation in the *gyrA* gene; similarly, none of the six Cip^s^ Nal^r^ isolates harbored this mutation in the *gyrA* gene. Isolates that were resistant to ciprofloxacin and/or nalidixic acid but had no C257T mutation in the *gyrA* gene showed MICs in the range of 4-32 mg/L for CIP and 32-256 mg/L for Nal.

The erythromycin-resistant strains were screened for the presence of 23S rRNA, *rplD, rplV* and *cmeRABC* locus mutations tentatively associated previously with erythromycin resistance in *Campylobacter*. Within the 23S rRNA gene of these strains, the A2074G mutation was not observed in any of the isolates and the A2075G mutation was identified in only one isolate displaying a high level of erythromycin resistance (>256 mg/L). Further analysis of the *rplD* and *rplV* genes (encoding the 50S ribosomal subunit proteins L4 and L22, respectively) identified several different potential amino acid substitutions (Table 3). The following predicted amino acid substitutions were observed in L4: V121A (two isolates), T177S (one isolate), M192I (two isolates), V196A (five isolates). For L22, more diverse amino acid substitutions were identified including: Q24R (one isolate), V65I (eight isolates), G74A (ten isolates), A103V (one isolate), T109S (ten isolates), A111E (ten isolates), and A114T (ten isolates). Using the erythromycin-sensitive *C. jejuni* strain NCTC 11168 as a “wild-type” reference strain, four *CmeR* alleles were identified among the erythromycin-resistant isolates (Table 3, Figure S1A). Additionally, sequencing of the *cme* RAIVS (*cmeR*-*cmeA* intervening sequence) region identified three alleles, with respect to strain NCTC 11168 (Table 3, Figure S1B). In addition to two SNPs, allele cmeRAIVS4 contained a six base insertion.

**Table 3:** Minimum Inhibitory concentrations (MICs), 23S rRNA gene mutations, ribosomal protein substitutions and *cmeRABC* locus alleles in 10 erythromycin-resistant *C. jejuni* isolates.

**Strain**	**Ery MIC (mg/L)**	**Mutation in 23S rRNA gene**	***ermB***	**Ribosomal protein polymorphisms**		***cmeR***	**RAIVS**
				**L4 mutation**	**L22 mutation**		
810	>256	A2075G	–	A196V	V65I, G74A, T109S, A111E, A114T	3	3
1062	>256	WT	–	A196V	V65I, G74A, T109S, A111E, A114T	WT	WT
1396	>256	WT	–	0	G74A, A103V, T109A, A111E, A114T	4	4
656	12	WT	–	0	Q24R, V65I, G74A, T109A, A111E, A114T	2	2
713	128	WT	–	A196V	V65I, G74A, T109S, A111E, A114T	NT	NT
783	32	WT	–	A196V	V65I, G74A, T109S, A111E, A114T	3	3
856	6	WT	–	M192I, V121A	G74A, T109A, A111E, A114T	2	2
872	>256	WT	–	0	V65I, G74A, T109S, A111E, A114T	WT	WT
1680	>256	WT	–	M192I, V121A, T177S	V65I, G74A, T109A A111E, A114T	2	2
2136	12	WT	–	A196V	V65I, G74A, T109S, A111E, A114T	5	4

*WT, wild type (with respect to C. jejuni strain NCTC 11168); NT, not tested; RAIVS, cmeRA intervening sequence. For cme locus alleles, see also Figure S1*.

### Association of MLST sequence types (STs) and clonal complexes (CCs) with antimicrobial resistance patterns and *gyrA* mutations

**Figure 1:**
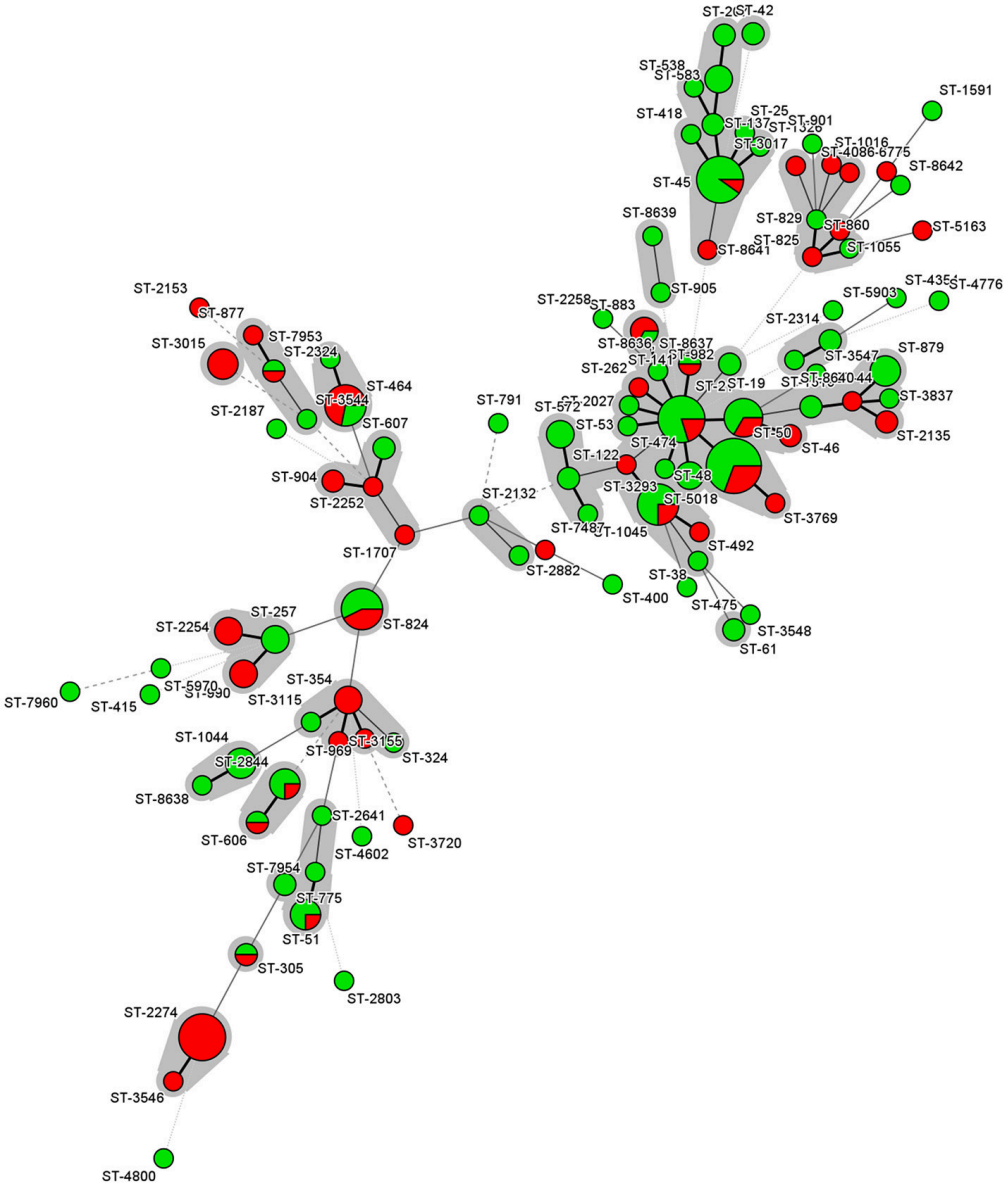
Minimum spanning tree of MLST data for the 204 *C. jejuni* isolates. Each node represents a particular ST, and the size of the circle is proportional to the number of isolates sharing the same ST. Inside the circles, the colors reflect the number of antimicrobial resistances. Green color represents isolates with less than 3 antimicrobial resistances while red color represents isolates with three or more resistances. Each individual sequence type is distinguished by separate circles and linked by lines indicating allelic variation. STs belonging to the same CC are color-shaded.

The analysis was restricted only on those STs and CCs represented by five or more isolates. The distribution of MICs for the different antibiotics among the sequence types revealed minor differences, except for strains from ST-824 (*p* = 0.016) and ST-2274 (*p* = 0.002), which were significantly more resistant to ciprofloxacin and nalidixic acid than strains from other STs (Figure 1). The distribution of MICs for the different antibiotics among the clonal complexes revealed that strains from CC-45 were more sensitive against ciprofloxacin (*p* < 0.001), tetracycline (*p* = 0.0201) and nalidixic acid (*p* = 0.047) than strains from other clonal complexes.

The statistical analyses revealed that three STs were significantly associated with the presence of *gyrA* mutations. These are ST-2274 (*p* = 0.001), and ST-824 (*p* = 0.02), which showed a higher proportion of isolates with *gyrA* mutations. On the other hand, ST-45 did not show any isolate with *gyrA* mutation (*p* = 0.01). The prevalence of *gyrA* mutations was also significantly linked to the clonal complex CC-574 (*p* = 0.02937), while all CC-45 (*p* = 0.047) isolates showed no *gyrA* mutations.

## Discussion

The rising trend of antimicrobial resistance among C*. jejuni* strains represents a serious public health concern. In recent years, many studies conducted worldwide reported high levels of resistance to ciprofloxacin and tetracycline, and emerging resistance to macrolides (Engberg et al., 2001; Luangtongkum et al., 2009). Therefore, continuous monitoring of resistance rates and mechanisms of resistance is crucial to combat the potential spread of AMR *C. jejuni* across the food chain. This study was conducted to provide better insight into the dynamics and molecular epidemiology of *C. jejuni* antibiotic resistance by characterizing 204 isolates from broiler carcasses obtained in Belgium over a decade (2006–2015). The majority of antimicrobials screened (all antimicrobials used except tetracycline) are critically important for human health, being classified in the Rank I by the World Health Organization (WHO, 2011).

In this study, approximately half of the isolates were resistant to nalidixic acid (53.9%), ciprofloxacin (53.4%) or tetracycline (47%). These levels of resistance are consistent with recent data obtained in another Belgian study carried out in 2007 that analyzed *C. jejuni* isolates from chicken meat (Habib et al., 2009). However, these resistance levels are moderately higher than those reported in other studies previously conducted in Belgium (Van Looveren et al., 2001; Mattheus et al., 2012). Mattheus et al. (2012) performed a surveillance analysis in poultry meat over the period 2004–2009, reporting the following resistance figures: 39.5% for nalidixic acid, 38.0% for ciprofloxacin and 40.8% for tetracycline. Similar findings were observed in 1998 in another study of AMR that included *C. jejuni* strains isolated from broilers, where rates of resistance to nalidixic acid, ciprofloxacin and tetracycline were 44.2, 44.2, and 34.4%, respectively (Van Looveren et al., 2001). The combination of these results reflects the relatively high resistance to these antimicrobials among Belgian *C. jejuni* isolates and a slight increase of AMR in *C. jejuni* over the last two decades in poultry production. Fluoroquinolones have commonly been used to treat infection in poultry production, while tetracyclines have been frequently used in animal production to treat infections and as growth promoters over the last 50 years (Giacomelli et al., 2014). The high resistance rates observed for these antimicrobials may be the consequence of their continuous overuse.

*C. jejuni* isolates that were resistant to ciprofloxacin and/or nalidixic acid were screened for the presence of a T86I amino acid substitution in the QRDR of the *gyrA* gene. The present results showed that this mutation was absent in some isolates that were resistant only to ciprofloxacin or nalidixic acid, and five isolates that showed simultaneous resistance to both antimicrobials. This observation is in agreement with previous reports that suggest that this substitution does not confer universal resistance to all quinolone antibiotics and concluded that quinolone resistance might also be attributed to other unknown resistance mechanisms (Dionisi et al., 2004; Corcoran et al., 2005; Bolton et al., 2013). This idea is further supported by the fact that although T86I mutations in the *gyrA* gene have been previously reported to be associated with high levels of resistance to nalidixic acid (MIC 64-256 mg/L) and ciprofloxacin (MIC 16-64 mg/L) (Engberg et al., 2001), we identified three Nal^r^ isolates (cj473, cj3408, cj2500), one Cip^r^ isolate (cj2940) and two Nal^r^ Cip^r^ isolates (cj1062, cj857) without the *gyrA* T86I mutation that nevertheless exhibited MICs within those ranges. Future studies will be directed to identify additional mutations, other than *gyrA* T86I, that also confer resistance to ciprofloxacin and/or nalidixic acid.

In this study, the low resistance rates to erythromycin and gentamicin are consistent with earlier reports in Belgian *C. jejuni* strains isolated from broiler meat. For instance, previously reported resistance levels to erythromycin and gentamicin were as low as 0.7 and 1.4% (Habib et al., 2009), 6.3 and 0% (Van Looveren et al., 2001), and 6 and 12.9% (Mattheus et al., 2012). These results are also in agreement with another European study performed in Italy (Giacomelli et al., 2014). Despite the common use of macrolides in poultry, for instance the use of tylosin in young birds (Giacomelli et al., 2014), the low resistance to erythromycin found in our study may be due to the slower development of erythromycin-resistant mutants during exposure to antibiotics, the reduced biological fitness of resistant mutants and/or their reduced ability to survive in the absence of selective pressure (Luangtongkum et al., 2009; Luangtonkum et al., 2012). The infrequent isolation of strains resistant to the aminoglycoside gentamicin might be attributed to the rare use of this antimicrobial agent as a prophylactic or therapeutic agent in broiler production systems (Rodrigo et al., 2007).

A2074G and A2075G mutations in region V of the 23S rRNA gene have been previously reported as prominent contributors to high-level macrolide resistance in *Campylobacter* (Niwa et al., 2001; Vester and Douthwaite, 2001; Haanperä et al., 2005; Corcoran et al., 2006; Caldwell et al., 2008). However, in this study, A2074G was not identified and A2075G was identified in only one isolate displaying high level erythromycin resistance. Indeed, the A2075G mutation was absent in five high-level erythromycin-resistant isolates (MIC > 128 mg/L). These findings are similar to previous observations from broiler flocks in Italy, where only 3.1% (1/36) of the macrolide-resistant *C. jejuni* isolates carried the A2075G mutation in the 23S rRNA gene and none harbored the A2074G mutation (Giacomelli et al., 2012). This observation could be due to a lower frequency of occurrence of these mutations in the 23S rRNA gene in *C. jejuni* when compared to *C. coli* as previously hypothesized by Giacomelli et al. (2012). The absence of this mutation among low-level erythromycin-resistant isolates was nonetheless not unexpected, as this mutation has been linked to high-level erythromycin resistance (Alonso et al., 2005). These results prompted us to investigate other possible alternative mechanisms of macrolide resistance, including modifications in the 50S ribosomal subunit proteins L4 and L22 and modifications at the *CmeABC* efflux pump locus.

As ribosomal components, the 50S subunit proteins L4 and L22 are highly conserved proteins in bacteria. Therefore, mutations in the genes encoding the L4 and L22 proteins affect the binding of macrolides to the 50S ribosomal subunit, resulting in macrolide resistance (Gibreel and Taylor, 2006; Belanger and Shryock, 2007). In *Campylobacter*, mutations in the large loop of the L4 protein (residues 55–77) and the L22 protein (residues 78–98) have been confirmed to be associated with macrolide resistance in various strains. In the present study, all mutations observed were found outside of the loop regions of the L4 or L22 proteins (Table 3). Furthermore, some of the identified mutations were previously identified both in resistant and susceptible isolates, for example V121A, A196V, A111E, and A114T in the L4 or L22 proteins (Corcoran et al., 2006). Thus, although it is possible that the ribosomal protein mutations observed here may contribute to macrolide resistance, it is likely that mutations external to the L4 and L22 proteins are the basis of the observed erythromycin resistance in these strains.

In *C. jejuni*, transcriptional regulation of the efflux pump operon *cmeABC* is carried out by the repressor *CmeR*, which binds specifically to an inverted repeat (IR) in the intervening sequence between *cmeR* and *cmeA* (Lin et al., 2005a). This IR overlaps the predicted −35 region in the *cmeABC* promoter (Lin et al., 2005b; see also Figure S1B). Lin et al. (2005b) demonstrated that a single bp deletion in the two deletion in the two bp IR spacer resulted in decreased *CmeR* binding with a concomitant elevation of fluoroquinolone resistance. In this study, mutations were identified in the *cmeR*-*cmeA* intergenic region; however one of the mutations, A58T in alleles cmeRAIVS2-3 (Figure S1B), might be expected to improve the IR and thus lead to enhanced *CmeR* binding. Only one of the mutations, a six bp insertion in allele cmeRAIVS4, is predicted to disrupt the IR, increasing the spacer between the two IR half sites from two to eight bp. However, this insertion would also increase the spacing between the predicted −35 and −16/−10 regions of P_cmeABC_ and thus might result in decreased transcription of *cmeABC*, despite the potential loss of repression by *CmeR*. Mutations were also observed in *CmeR* (Table 3, Figure S1A); however, the effect of these mutations on macrolide resistance remains to be determined, especially as it is unknown how these *CmeR* alleles interact with the RAIVS IR region. Taken together with the results of the 23S rRNA and L4/L22 analyses, the genetic basis of macrolide resistance in the ten strains identified in this study is largely unknown. Additional research will be necessary, potentially requiring whole genome sequencing and further analysis of *CmeR* binding to P_cmeABC_, in order to determine the cause or causes of macrolide resistance in these strains.

Previous studies have observed an association between some sequence types (STs) or clonal complexes (CCs), in *C. jejuni* isolated from different sources, and resistance to quinolones and tetracycline, suggesting a local clonal expansion of resistance phenotypes arising from the use of antimicrobials in different animal and human settings (Habib et al., 2009; Cody et al., 2012; Kittl et al., 2013; Kovač et al., 2014, 2015; Klein-Jöbstl et al., 2016). The correlation between CC-45 and susceptibility to quinolones and tetracycline observed in this study is in agreement with a study conducted by Habib et al. (2009) on *C. jejuni* isolates from chicken meat preparations in Belgium. To the best of our knowledge, this is the first study that reports an association of ST-824 and ST-2274 with resistance to ciprofloxacin and nalidixic acid. Interestingly, the correlation between ST-2274 and the CIP-NAL-TET AMR profile was notable. Considering that ST-2274 is among the most common STs found in Belgium (Elhadidy et al., 2018), these results suggest that ST-2274 is among the genotypes mainly responsible for the spread of this AMR pattern.

In conclusion, this study examined the mechanisms and the clonal expansion of antimicrobial resistance among *C. jejuni* strains isolated from broiler carcasses in Belgium. The previously-reported resistance mechanisms monitored in this study were not found in all resistant isolates, suggesting that resistance to the tested antibiotics might be attributed to other unknown resistance mechanisms.

## Author contributions

ME conceived and designed the study, performed the tests, analyzed and interpreted the data, and wrote the manuscript. WM performed all sequencing experiments. WM, AD, KD, and NB substantially contributed in analysis of the results, and revision of the manuscript; AÁ-O, and HA conducted the data analysis and contributed to the writing of the manuscript. All authors read and approved the final manuscript.

### Conflict of interest statement

The authors declare that the research was conducted in the absence of any commercial or financial relationships that could be construed as a potential conflict of interest.

## Supplementary material

Figure S1 Allelic variation within *CmeR* and the *cmeR*-*cmeA* intervening sequence. For the erythromycin-resistant isolates, the predicted *CmeR* protein sequences and the RAIVS (*cmeR*-*cmeA* intervening sequence) nucleotide sequences were determined. Relative to the erythromycin-sensitive *C. jejuni* strain NCTC 11168, four *CmeR* and three RAIVS alleles were identified. These alleles (and the respective sequences from strain NCTC 11168) were aligned using MegAlign (v. 8.0, DNASTAR, Madison, WI). Using the sequences of strain NCTC 11168 as “wild-type,” amino acid substitutions within the *CmeR* alleles **(A)** and nucleotide changes within the RAIVS alleles **(B)** are boxed. Half sites of the inverted repeat (IR) are shaded in gray. Predicted−35, −16, and−10 regions are labeled. Promoter and IR regions are as described in Lin et al. (2005a).
