# Supplementary figures and images for: Genetic Basis and Clonal Population Structure of Antibiotic Resistance in Campylobacter jejuni Isolated From Broiler Carcasses in Belgium
Source: Front Microbiol. 2018 May 17;9:1014. doi: 10.3389/fmicb.2018.01014 (PMC5966580; doi:10.3389/fmicb.2018.01014)

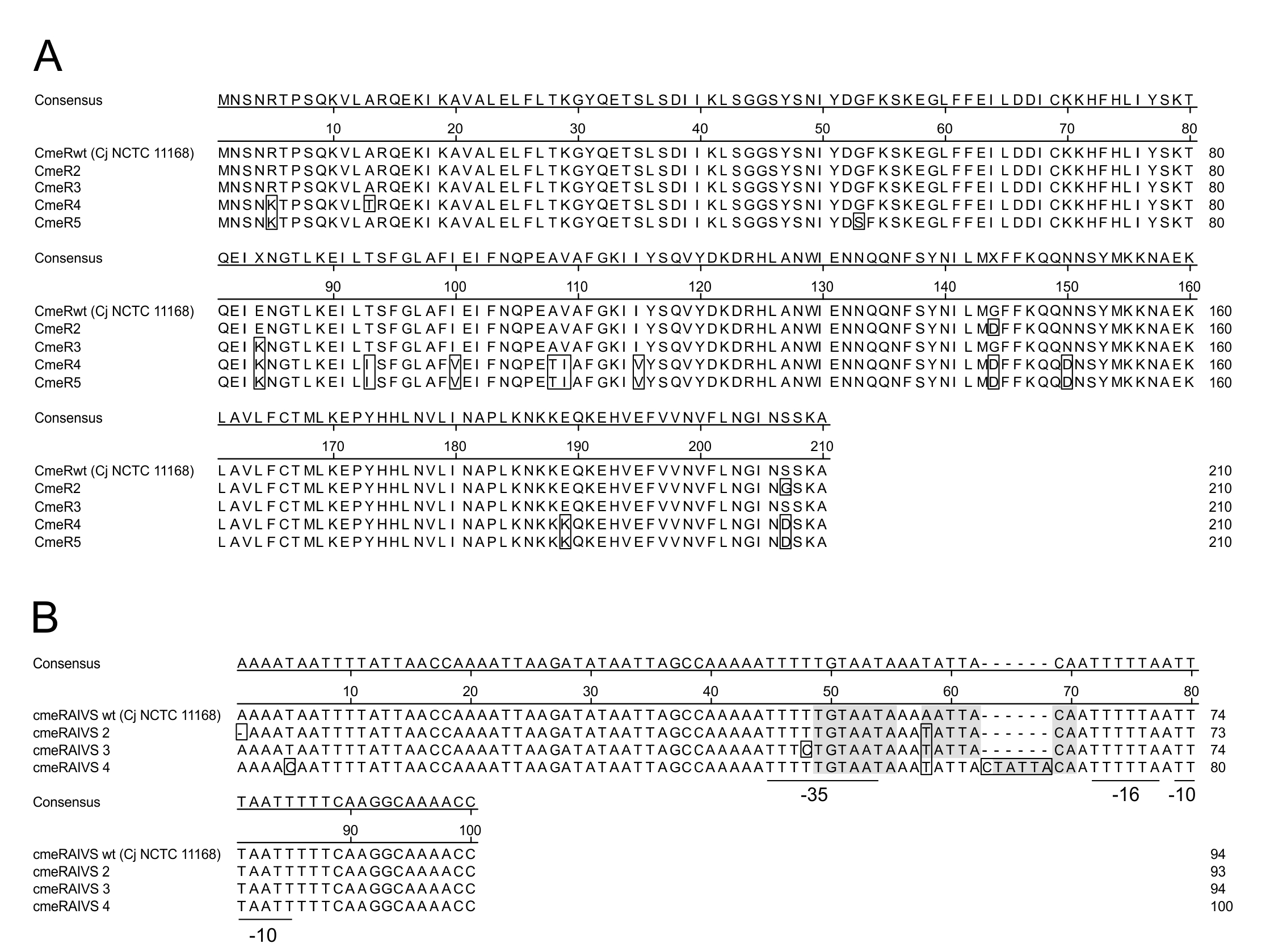

Supplement: Figure S1 — Allelic variation within CmeR and the cmeR-cmeA intervening sequence. For the erythromycin-resistant isolates, the predicted CmeR protein sequences and the RAIVS (cmeR-cmeA intervening sequence) nucleotide sequences were determined. Relative to the erythromycin-sensitive C. jejuni strain NCTC 11168, four CmeR and three RAIVS alleles were identified. These alleles (and the respective sequences from strain NCTC 11168) were aligned using MegAlign (v. 8.0, DNASTAR, Madison, WI). Using the sequences of strain NCTC 11168 as “wild-type,” amino acid substitutions within the CmeR alleles (A) and nucleotide changes within the RAIVS alleles (B) are boxed. Half sites of the inverted repeat (IR) are shaded in gray. Predicted−35, −16, and−10 regions are labeled. Promoter and IR regions are as described in Lin et al. (2005a). [file Image_1.JPEG]
